# Characterizing Y-STRs in the Evaluation of Population Differentiation Using the Mean of Allele Frequency Difference between Populations

**DOI:** 10.3390/genes11050566

**Published:** 2020-05-19

**Authors:** Yuxiang Zhou, Yining Yao, Baonian Liu, Qinrui Yang, Zhihan Zhou, Chengchen Shao, Shilin Li, Qiqun Tang, Jianhui Xie

**Affiliations:** 1 Department of Forensic Medicine, School of Basic Medical Sciences, Fudan University, Shanghai 200032, China; 16111010009@fudan.edu.cn (Y.Z.); 19111010077@fudan.edu.cn (Y.Y.); 17111010084@fudan.edu.cn (B.L.); 17111010017@fudan.edu.cn (Q.Y.); 19211010067@fudan.edu.cn (Z.Z.); 07301010122@fudan.edu.cn (C.S.); 2 Ministry of Education Key Laboratory of Contemporary Anthropology and State Key Laboratory of Genetic Engineering, Collaborative Innovation Center for Genetics and Development, School of Life Sciences, Fudan University, Shanghai 200433, China; lishilin@fudan.edu.cn; 3 Department of Biochemistry and Molecular Biology, School of Basic Medical Sciences, Fudan University, Shanghai 200032, China; qqtang@shmu.edu.cn

**Keywords:** Y-STR, population differentiation, allele frequency difference, pairwise genetic distance, multidimensional scaling

## Abstract

Y-chromosomal short tandem repeats (Y-STRs) are widely used in human research for the evaluation of population substructure or population differentiation. Previous studies show that several haplotype sets can be used for the evaluation of population differentiation. However, little is known about whether each Y-STR in these sets performs well during this procedure. In this study, a total of 20,927 haplotypes of a Yfiler Plus set were collected from 41 global populations. Different configurations were observed in multidimensional scaling (MDS) plots based on pairwise genetic distances evaluated using a Yfiler set and a Yfiler Plus set, respectively. Subsequently, 23 single-copy Y-STRs were characterized in the evaluation of population differentiation using the mean of allele frequency difference (mAFD) between populations. Our results indicated that DYS392 had the largest mAFD value (0.3802) and YGATAH4 had the smallest value (0.1845). On the whole, larger pairwise genetic distances could be obtained using the set with the top fifteen markers from these 23 single-copy Y-STRs, and clear clustering or separation of populations could be observed in the MDS plot in comparison with those using the set with the minimum fifteen markers. In conclusion, the mAFD value is reliable to characterize Y-STRs for efficiency in the evaluation of population differentiation.

## 1. Introduction

Over the past decade, Y-chromosomal short tandem repeats (Y-STRs) have been widely used in human research and forensic practice to supplement information retrieved from autosomal DNA profiling. Y-STRs can be applied for acquiring male genotypes from stains of unbalanced male/female DNA mixture [1,2,3], for testing paternal kinship [3,4], for determining paternal biogeographic ancestry of missing persons [3] and so on. While the primary Y-STR set, namely the minimal haplotype [5], allows the genotyping of only 9 loci, the Yfiler Plus PCR amplification kit, released in 2014, can simultaneously detect 17 Y-STR loci adopted in the Yfiler kit and 10 new Y-STR loci (DYS481, DYS460, DYS533, DYS449, DYS576, DYS627, DYS518, DYS570 and DYF387S1). As for these new markers, there are seven rapidly mutating Y-STR loci and three highly polymorphic Y-STR loci [6].

Because of the male-specific inheritance pattern and haploid nature of the Y chromosome, it is more sensitive to genetic drift and founder effect for Y-linked markers than for autosomal ones. Y-STRs are widely used for the evaluation of population substructure or population differentiation by testing pairwise genetic distances. The clustering or separation of populations can further be visualized using multidimensional scaling (MDS) analysis based on the similarity of pairwise genetic distances between populations. Previous studies show that several haplotype sets, such as the minimal haplotype set, Yfiler set, PowerPlex Y23 set and Yfiler Plus set, can be used in the evaluation of population differentiation [7,8,9,10,11]. In fact, compared with the original population, allelic configuration of Y-STRs in newly established populations might vary from locus to locus. Therefore, the allelic configurations of Y-STRs in newly established populations might be similar to or dramatically different from that in the original population. Generally, the single-locus genetic diversity and haplotype diversity are used to evaluate discrimination capacity of Y-STR markers and marker sets in populations, respectively. Recently, Shannon’s equivocation was reported to quantify the allelic association between Y-STRs to obtain maximally discriminatory marker sets [12]. In contrast, investigations are rarely conducted to characterize Y-STRs for efficiency in the evaluation of population differentiation.

In this study, we characterized 23 single-copy Y-STRs adopted in a Yfiler Plus set in the evaluation of population differentiation using the mean of allele frequency difference (mAFD) between populations. Haplotype profiles of the Yfiler Plus set were compiled from 41 global populations, and an evaluation of population differentiation using the Yfiler set and Yfiler Plus set was performed. Further, the mAFD values of 23 Y-STR loci between 41 populations were calculated, respectively, and the reliability of the mAFD to characterize Y-STRs in the evaluation of population differentiation was estimated based on pairwise genetic distances and MDS analysis from a series of fifteen-marker sets.

## 2. Materials and Methods

### 2.1. Data-Set Collection

A total of 41 populations with haplotypes of 27 Y-STR loci in a Yfiler Plus set were collected (Table S1). To facilitate the subsequent analysis, haplotypes with null alleles, intermediate alleles, duplicated or triplicatd alleles were removed, which resulted in a final collection of 20,927 haplotypes. Because DYS389I is a part of DYS389II, each DYS389I allele was subtracted from the DYS389II allele to obtain another part of DYS389II.

### 2.2. Calculation of the Mean of Allele Frequency Difference

The allele frequency difference (AFD) of each Y-STR locus between two populations was defined as follows according to previous reports [13]:

$$\mathrm{AFD}=\frac{1}{2}\sum_{i=1}^{n}\left|\left(f_{i1}-f_{i2}\right)\right|,$$

where *n* is the total number of alleles at a locus, and *f_i_*-terms denote the frequency of the *i*th allele in the two populations. As for every Y-STR, the mean of the AFD across all population pairs was obtained based on the AFD for every population pair. Here, the mAFD values of every single-copy Y-STRs were computed using an in-house Python script.

### 2.3. The Evaluation of Population Differentiation

The evaluation of population differentiation using a Yfiler set and a Yifler Plus set was carried out. Pairwise genetic distances of these two marker sets were gauged using Arlequin v3.5 [14], and a line chart was produced simultaneously. In the assessment of pairwise genetic distances, *p* values were calculated at a significant level of 0.05 using 10,000 permutations. In addition, MDS analysis estimating similarity quantitatively among populations was performed with the software IBM SPSS**^®^** Statistics version 22 (IBM Corp., Armonk, NY, USA). The output of MDS is a plot that reveals the relational structures of objects, where similar objects cluster together, and dissimilar ones are far from each other.

For validating the efficacy of the mAFD in the evaluation of population differentiation, nine marker sets containing 15 Y-STRs were constructed along with the stepwise reduction of mAFD value (step size: one marker) using the 23 Y-STRs evaluated. Pairwise genetic distances between populations for these Y-STR sets were quantified and compared. To visualize the differences in pairwise genetic distances between populations, MDS analysis of all marker sets was conducted.

## 3. Results and Discussion

### 3.1. Evaluation of Population Differentiation Using a Yfiler Set and a Yfiler Plus Set

**Figure 1:**
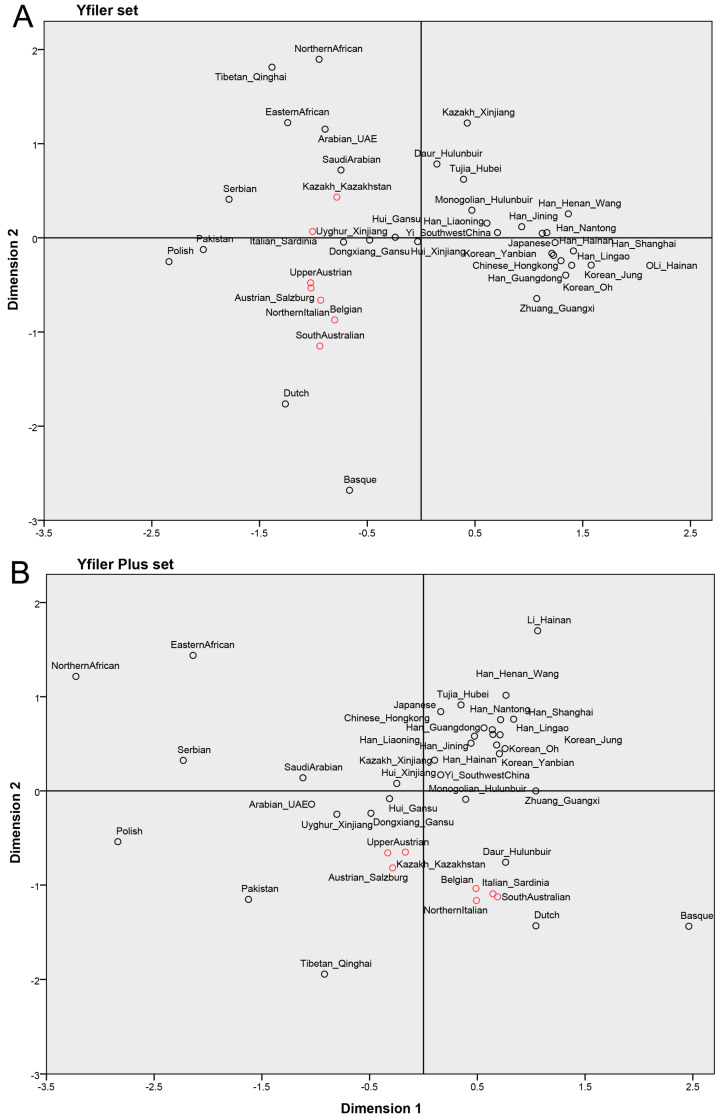
The multidimensional scaling (MDS) plots obtained based on pairwise genetic distances between populations with a Yfiler set (**A**) and a Yfiler Plus set (**B**), respectively.

**Figure 2:**
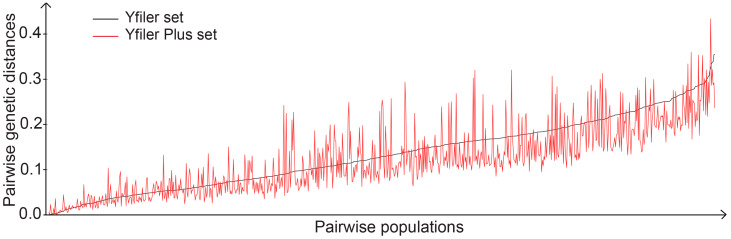
Pairwise populations are ranked in an ascending order of pairwise genetic distances with a Yfiler set and are set as the horizontal axis. Pairwise genetic distances with a Yfiler set and a Yfiler Plus set are indicated, respectively.

To evaluate the population differentiation, the pairwise genetic distances between 41 populations were calculated based on a Yfiler set and Yfiler Plus set, respectively, and population differentiation was visualized in a MDS plot. On the whole, the distribution of populations was in accordance with the biogeographic pattern based on pairwise genetic distances tested using a Yfiler set or a Yfiler Plus set (Figure 1A,B). However, the difference could be observed between these two MDS plots. For example, the distribution of seven populations marked in red circles in Figure 1A,B, including Kazakh_Kazakhstan, UpperAustrian, Austrian_Salzburg, Belgian, Italian_Sardinia, NorthernItalian and SouthAustralian, was scattered in the MDS plot of the Yfiler set, while two clusters could be clearly observed in the MDS plot of the Yfiler Plus set (Figure 1A,B). Generally, pairwise genetic distances between populations decrease with an increasing number of Y-STRs in a haplotype set (Figure 2) [15,16]. However, approximately one-third of pairwise genetic distances evaluated based on the Yfiler Plus set showed an increase compared with those using the Yfiler set (Tables S2 and S3), which might contribute to the clustering or separation of populations in MDS analysis.

In this study, the same population sets were employed to evaluate population differentiation using a Yfiler set and a Yfiler Plus set, which can reduce the bias from sampling using different population sets. The Yfiler Plus set seems to more clearly cluster or separate populations than the Yfiler set in MDS analysis (Figure 1A,B), which shows that the similarity of the population can be different using different marker sets for the evaluation of population differentiation even though the same datasets were used. Therefore, these results imply that each Y-STRs might present different genetic diversity across these populations, which leads to an increase or decrease of pairwise genetic distances when adding new Y-STR loci into a Yfiler set.

### 3.2. Assessment of the mAFD Values of Y-STRs

To characterize Y-STRs in the evaluation of population differentiation, the mAFD values of 23 Y-STRs were calculated as described in Material and Methods section. As shown in Table 1, the largest mAFD value was observed at DYS392 (mAFD = 0.3802), followed by DYS438 (mAFD = 0.3507) and DYS635 (mAFD = 0.3379). The smallest two ones were at YGATAH4 (mAFD = 0.1845) and DYS391 (mAFD = 0.1891). Within the top 15 loci, 9 loci were from Yfiler set and 6 loci were from 10 newly added loci in the Yfiler Plus set (Table 1). While the values of mAFD vary from 0 to 1 theoretically, according to the evaluation formula, none was above 0.4 in this study, suggesting that the genetic variance between human populations was not remarkable for a single locus. The mAFD should imply the degree of variation for one Y-STR in populations and might represent the power that contributes to the haplotype set in the evaluation of population differentiation. It should be noted that the value of the mAFD mostly relied on tested populations and could be varied because of the number and the distribution patterns of populations tested. In this study, a set of global populations was sampled to diminish the deviation in the evaluation of the mAFD of every Y-STR.

In this study, the mAFD is described to evaluate the variance of genetic markers in populations, which is somewhat similar to Rogers’ distance [17,18]. Rogers’ distance is used to measure genetic distance between two populations using the variance of allelic frequency of genetic markers. In contrast, the mAFD aims to evaluate the variance of genetic markers across populations rather than genetic distance between populations. The evaluation of Rogers’ distance can be impaired due to the increased number of alleles [18]. As for the mAFD, it represents an overall diversity across all tested populations. Therefore, the order of these markers ranked using mAFD values should not be drastically modified by the addition of several new populations.

**Figure 3:**
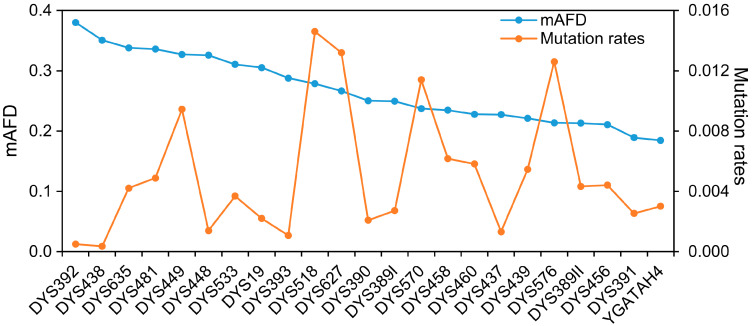
The mAFD values and mutation rates of 23 Y-STRs. Blue line: mAFD; Orange line: Mutation rates.

The difference of allelic frequency between populations might be affected by many factors, such as founder effect, genetic drift and mutation rate. The mutation events of each Y-STR occur independently in the population, although the evaluation of genetic distances is based on the Y-STR haplotypes. A low mutation rate for Y-STRs might make the difference of allelic frequency more sensitive to genetic drift and founder effect. In contrast, a high mutation rate for Y-STRs might help to rapidly establish a permanent allelic configuration in the population. In this study, the mutation rate did not show a close correlation with the mAFD (Figure 3). Similarly, the single-locus genetic diversity obtained from all tested populations did not show a close correlation with the mAFD (data not shown). Therefore, genetic drift and founder effect might be important factors for the mAFD.

### 3.3. Analysis of Y-STR Marker Sets in the Population Differentiation

**Figure 4:**
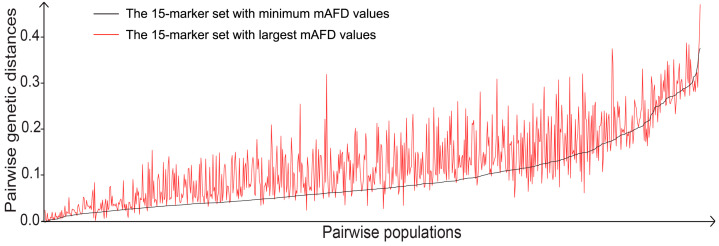
Pairwise populations are ranked in an ascending order of pairwise genetic distances with the 15-marker set of the minimum mAFD values, and are set as the horizontal axis. Pairwise genetic distances with the 15-marker set of the largest mAFD values and of the minimum mAFD values are indicated, respectively.

To validate the efficacy of the mAFD in the evaluation of population differentiation, 23 Y-STR markers were ranked in a descending order based on the mAFD, and nine sets were established using a sliding-set method with a set of 15 markers and a step size of one marker. The pairwise genetic distances calculated using these sets could be varied (Tables S4–S12), and the tendency was observed that pairwise genetic distances increased with the application of the marker sets with larger mAFD values (Figure 4). It should be noted that a small number of pairwise genetic distances was decreased when a marker with a low mAFD value was replaced by one with a high mAFD value, which suggests that the replacement with the marker with the high mAFD value could result in a lower genetic diversity of haplotypes between two populations.

**Figure 5:**
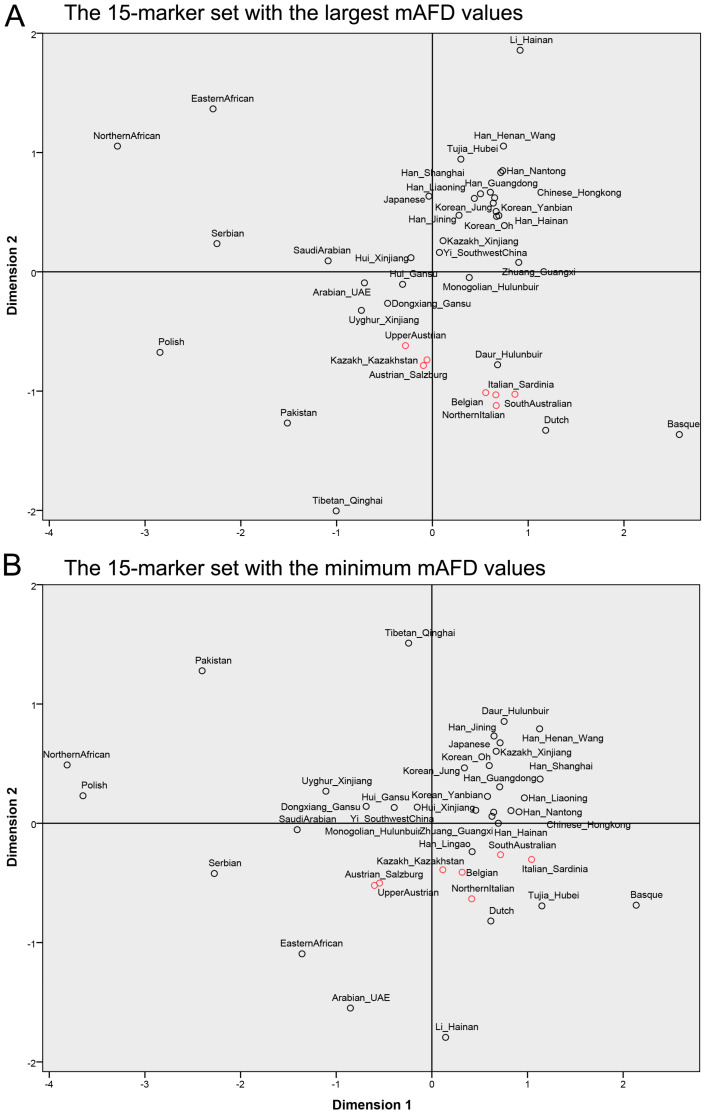
The MDS plots obtained based on pairwise genetic distances between populations with the marker sets of the largest (**A**) and the minimum (**B**) mAFD values, respectively.

To visualize population differentiation, MDS analysis was performed based on pairwise genetic distances obtained using the sets with the top fifteen markers and the minimum fifteen markers, respectively. As shown in Figure 5A, the set with the top fifteen markers could clearly cluster or separate tested populations according to biogeographic patterns, which was similar to the performance of the Yfiler Plus set. In contrast, the set with the minimum fifteen markers did not effectively differentiate these tested populations (Figure 5B). It might be that genetic distances obtained using the set with the top fifteen markers have a relatively slight increase tendency between close populations and a relatively large increase tendency between distant populations, in comparison to those obtained using the set with the minimum fifteen markers (Figure 4). Altogether, these results demonstrate that the use of the mAFD to characterize Y-STRs in the evaluation of population differentiation is reliable.

## 4. Conclusions

Genetic distance is defined as the extent of genetic differentiation between populations or species. Previous studies demonstrate the capacity of several haplotype sets in the evaluation of population differentiation through genetic distance, and little attention has been paid to the selection of genetic markers. Unlike autosomal markers, which can reach Hardy–Weinberg equilibrium, Y-STRs are paternally inherited. Currently, it remains unknown whether a Y-STR locus in a haplotype is suitable for the evaluation of population differentiation. Although the method based on the mAFD value for the evaluation of single Y-STR is relatively simple in this study, our results show that the mAFD is suitable for characterizing Y-STRs in the evaluation of population differentiation. In the future, the introduction of more global populations can improve the evaluation of the mAFD value for each Y-STR.

## Figures and Tables

**Table 1:** Allele frequency difference (mAFD) values and mutation rates of 23 Y-chromosomal short tandem repeats (Y-STRs).

	Loci	mAFD	Mutation Rates ^#^	Binomial 95%CI
1	**DYS392**	0.3802	4.96 × 10^−4^	2.14 × 10^−4^–9.77 × 10^−4^
2	**DYS438**	0.3507	3.51 × 10^−4^	0.96 × 10^−4^–8.99 × 10^−4^
3	**DYS635**	0.3379	4.21 × 10^−3^	2.97 × 10^−3^–5.80 × 10^−3^
4	DYS481	0.3359	4.88 × 10^−3^	2.52 × 10^−3^–8.51 × 10^−3^
5	DYS449	0.3271	9.44 × 10^−3^	5.78 × 10^−3^–1.45 × 10^−2^
6	**DYS448**	0.3257	1.39 × 10^−3^	6.92 × 10^−4^–2.48 × 10^−3^
7	DYS533	0.3104	3.68 × 10^−3^	1.68 × 10^−3^–6.97 × 10^−3^
8	**DYS19**	0.3053	2.20 × 10^−3^	1.55 × 10^−3^–3.04 × 10^−3^
9	**DYS393**	0.2878	1.07 × 10^−3^	6.11 × 10^−4^–1.74 × 10^−3^
10	DYS518	0.2786	1.46 × 10^−2^	9.86 × 10^−3^–2.08 × 10^−2^
11	DYS627	0.2666	1.32 × 10^−2^	8.95 × 10^−3^–1.88 × 10^−2^
12	**DYS390**	0.2504	2.08 × 10^−3^	1.44 × 10^−3^–2.91 × 10^−3^
13	**DYS389I**	0.2494	2.72 × 10^−3^	1.96 × 10^−3^–3.69 × 10^−3^
14	DYS570	0.2372	1.14 × 10^−2^	7.67 × 10^−3^–1.62 × 10^−2^
15	**DYS458**	0.2345	6.17 × 10^−3^	4.57 × 10^−3^–8.15 × 10^−3^
16	DYS460	0.2278	5.82 × 10^−3^	2.80 × 10^−3^–1.69 × 10^−2^
17	**DYS437**	0.2273	1.32 × 10^−3^	7.39 × 10^−4^–2.18 × 10^−3^
18	**DYS439**	0.2211	5.46 × 10^−3^	4.19 × 10^−3^–6.99 × 10^−3^
19	DYS576	0.2136	1.26 × 10^−2^	8.86 × 10^−3^–1.73 × 10^−2^
20	**DYS389II**	0.2131	4.33 × 10^−3^	3.34 × 10^−3^–5.51 × 10^−3^
21	**DYS456**	0.2106	4.41 × 10^−3^	3.07 × 10^−3^–6.12 × 10^−3^
22	**DYS391**	0.1891	2.53 × 10^−3^	1.82 × 10^−3^–3.43 × 10^−3^
23	**YGATAH4**	0.1845	3.01 × 10^−3^	1.98 × 10^−3^–4.38 × 10^−3^

^#^ The data of mutation rates is from the Y Chromosome Haplotype Reference Database (YHRD) website (release 61, Jun 2019) [19]. Y-STRs in the Yfiler set are indicated in bold.

## Supplementary Materials

The following are available online at https://www.mdpi.com/2073-4425/11/5/566/s1, Table S1: Size and residency of population samples, Table S2: pairwise genetic distances (RST, below the diagonal) and corresponding p values (above the diagonal) for a Yfiler set between 41 populations, Table S3: Pairwise genetic distances (RST, below the diagonal) and corresponding p values (above the diagonal) for a YfilerPlus set between 41 populations, Table S4: Pairwise genetic distances (RST, below the diagonal) and corresponding p values (above the diagonal) between 41 populations for the set composed of loci ranking from 1 to 15 in Table 1, Table S5: Pairwise genetic distances (RST, below the diagonal) and corresponding p values (above the diagonal) between 41 populations for the set composed of loci ranking from 2 to 16 in Table 1, Table S6: Pairwise genetic distances (RST, below the diagonal) and corresponding p values (above the diagonal) between 41 populations for the set composed of loci ranking from 3 to 17 in Table 1, Table S7: Pairwise genetic distances (RST, below the diagonal) and corresponding p values (above the diagonal) between 41 populations for the set composed of loci ranking from 4 to 18 in Table 1, Table S8: Pairwise genetic distances (RST, below the diagonal) and corresponding p values (above the diagonal) between 41 populations for the set composed of loci ranking from 5 to 19 in Table 1, Table S9: Pairwise genetic distances (RST, below the diagonal) and corresponding p values (above the diagonal) between 41 populations for the set composed of loci ranking from 6 to 20 in Table 1, Table S10: Pairwise genetic distances (RST, below the diagonal) and corresponding p values (above the diagonal) between 41 populations for the set composed of loci ranking from 7 to 21 in Table 1, Table S11: Pairwise genetic distances (RST, below the diagonal) and corresponding p values (above the diagonal) between 41 populations for the set composed of loci ranking from 8 to 22 in Table 1, Table S12: Pairwise genetic distances (RST, below the diagonal) and corresponding p values (above the diagonal) between 41 populations for the set composed of loci ranking from 9 to 23 in Table 1.
